# AAV-mediated expression of NFAT decoy oligonucleotides protects from cardiac hypertrophy and heart failure

**DOI:** 10.1007/s00395-021-00880-w

**Published:** 2021-06-04

**Authors:** Anca Remes, Andreas H. Wagner, Nesrin Schmiedel, Markus Heckmann, Theresa Ruf, Lin Ding, Andreas Jungmann, Frauke Senger, Hugo A. Katus, Nina D. Ullrich, Norbert Frey, Markus Hecker, Oliver J. Müller

**Affiliations:** 1 Department of Internal Medicine III, University Hospital Schleswig-Holstein and University of Kiel , Arnold-Heller-Str. 3 , Kiel, Germany; 2 Internal Medicine III, University Hospital Heidelberg, Heidelberg, Germany; 3 Institute of Physiology and Pathophysiology, Heidelberg University, Heidelberg, Germany; 4 German Centre for Cardiovascular Research , Partner Site Hamburg/Kiel/Lübeck , Kiel, Germany

**Keywords:** Heart failure, Cardiac hypertrophy, Transcription factor, NFAT, Decoy oligonucleotide, Adeno-associated virus

## Abstract

Previous studies have underlined the substantial role of nuclear factor of activated T cells (NFAT) in hypertension-induced myocardial hypertrophy ultimately leading to heart failure. Here, we aimed at neutralizing four members of the NFAT family of transcription factors as a therapeutic strategy for myocardial hypertrophy transiting to heart failure through AAV-mediated cardiac expression of a RNA-based decoy oligonucleotide (dON) targeting NFATc1-c4. AAV-mediated dON expression markedly decreased endothelin-1 induced cardiomyocyte hypertrophy in vitro and resulted in efficient expression of these dONs in the heart of adult mice as evidenced by fluorescent in situ hybridization. Cardiomyocyte-specific dON expression both before and after induction of transverse aortic constriction protected mice from development of cardiac hypertrophy, cardiac remodeling, and heart failure. Singular systemic administration of AAVs enabling a cell-specific expression of dONs for selective neutralization of a given transcription factor may thus represent a novel and powerful therapeutic approach.

**Supplementary Information:**

The online version contains supplementary material available at 10.1007/s00395-021-00880-w.

## Introduction

Although treatment options have significantly improved prognosis and quality of life of patients diagnosed with heart failure, current therapies at best offer reduced disease progression but not a real cure [30]. Cardiac hypertrophy is initiated by various stress signals, such as growth factors and pressure or volume overload [12]. Although this state is compensatory by temporarily preserving cardiac output, sustained pathological myocardial hypertrophy is associated with increased risk of heart failure, arrhythmias and sudden death [12, 42].

Myocardial hypertrophic growth is a highly intricate process governed by activation of the calcineurin-NFAT signaling pathway [32, 45]. Following dephosphorylation by calcineurin, transcriptional activation of the NFAT family members c1 to c4 regulates central processes leading to heart failure, such as myocyte growth, extracellular matrix deposition and re-activation of the fetal gene program [17, 41]. A number of studies have delineated the major importance of NFAT in the development of cardiac hypertrophy. Cardiac-specific activation of the calcineurin-NFAT pathway was sufficient to induce myocardial hypertrophy [33], whereas both pharmacological inhibition [24] and genetic deletion of NFATc2 and c3 [2, 46] alleviated pathological remodeling in animal models. Therefore, reducing the transcriptional activity of NFAT could represent a valuable treatment option for cardiac hypertrophy and heart failure.

One approach of interfering with abnormal activity of transcription factors for therapeutic purposes is represented by decoy oligodeoxynucleotides (dODNs). These are short (10–25 bp) sequences of double-stranded DNA mimicking the consensus binding site of the target transcription factor, which upon cellular entry specifically neutralize it and hence inhibit transcription of its target genes [15]. These nucleic acid-based drugs have been proven to be safe and effective in various preclinical and clinical trials focusing on diverse disease models [29]. Until now, the most frequent route of administration employed is local delivery, which requires sustained application of the active dODNs [8, 9]. In terms of future therapy and depending on the target organ, this approach is not always feasible.

Here, we show that single injection of an adeno-associated viral vector (AAV9) is sufficient to express a decoy hairpin RNA oligonucleotide (dON) neutralizing NFATc1-c4 in cardiomyocytes in vivo. This singular treatment before or after subjecting mice to transverse aortic constriction (TAC) was associated with a dramatic improvement in cardiac function and reduced remodeling, and therefore broaden the paradigm of decoy oligo(deoxy)nucleotides as nucleic acid-based therapeutics.

## Materials and methods

### Primary cardiomyocytes isolation and culture

Primary neonatal rat cardiomyocytes (NRVCMs) were isolated according to previously established protocols [22]. Experiments were executed under the guidelines from Directive 2010/63/EU of the European Parliament on the protection of animals used for scientific purposes with approval of the local authorities in Kiel (permission number 1085). In brief, left ventricles from 2-day-old Wistar rats were harvested and digested in the presence of 0.6 mg/mL pancreatin (Sigma-Aldrich, Munich, Germany) and 0.5 mg/mL collagenase II (Worthington Biochemical Corporation, Lakewood, USA) at 37 °C. The resulting cell suspension was filtered through a cell strainer and afterwards centrifugation on a Percoll gradient (GE Healthcare, Chicago, USA) was performed to ensure separation from fibroblasts. Next, NRVCMs were cultured in complete DMEM medium containing 10% fetal bovine serum, 2 mmol/L L-glutamine, 100 U/mL penicillin, and 100 μg/mL streptomycin (Thermo Fischer Scientific, Darmstadt, Germany).

### DNA dODN technology

DNA-based dODNs were designed to form a hairpin structure by intramolecular hydrogen bond formation and to contain the promoter binding site of NFAT1-4 (Biomers, Ulm, Germany). A 5′-Atto-590 labeled dODN was used for assessment of cellular uptake efficiency. The sequences of the dODNs used in our study are as follows: hpNFAT cons dODN: 5′-GAGTGGAAACATACAGCCACTGAAACAGTGGCTGTATGTTTCCACTC-3′ and hpNFAT mut dODN: 5′-GAGCTTAAACATACAGCCACTGAAACAGTGGCTGTATGTTTCCACTC-3′. Decoys were dissolved in sterile TEN buffer to a concentration of 500 μmol/L, incubated at 95 °C for 5 min, followed by gradual cooling down, to induce hybridization of complementary base pairs and hairpin structure formation. Successful hybridization was proven by agarose gel electrophoresis.

### AAV production

Sequences of hpNFAT cons and mut dONs were synthesized (GeneArt, Thermo Fischer Scientific, Darmstadt, Germany) and cloned between XhoI and SalI restriction sites of the dON-generating vector, as previous described [27]. The gene synthesis sequences are presented below: hpNFATcons dON: 5′-AGGCGCCCTGCAATATTTGCATGTCGCTATGTGTTCTGGGAAATCACCATAAACGTGAAATGTCTTTGGATTTGGGAATCTTATAAGTTCTGTATGAGACCACAGTCGACGAGTGGAAACATACAGCCACTGAAACAGTGGCTGTATGTTTCCACTCCACCGCAGTTTCGACCTCGAGA-3′; hpNFATmut dON: 5′-AGGCGCCCTGCAATATTTGCATGTCGCTATGTGTTCTGGGAAATCACCATAAACGTGAAATGTCTTTGGATTTGGGAATCTTATAAGTTCTGTATGAGACCACAGTCGACGAGCTTAAACATACAGCCACTGAAACAGTGGCTGTATGTTTCCACTCCACCGCAGTTTCGACCTCGAGA-3′. AAV6 and AAV9 vectors were produced and purified as described elsewhere [16].

### Decoy ODN and AAV treatment

Decoy ODNs were added in serum-free cell culture medium to a concentration of 10 µmol/L prior to ET-1 stimulation. AAV transduction was conducted at a M.O.I. of 10^5^ vg/cell.

### Fluorescent in situ hybridization

The generation of hpNFAT RNA dONs following AAV9 transduction was verified in 5 µm myocardial frozen sections by fluorescent in situ hybridization according to standard protocols [47]. A molecular beacon with complementary sequence to the dON was used as a probe (Biomers, Ulm, Germany).

### Quantitative real-time PCR

Total RNA was extracted from adherent cells or tissue using RNeasy Mini Kit (Qiagen, Hilden, Germany) following the manufacturer’s instructions. First-strand synthesis of cDNA was completed using Omniscript Reverse Transcriptase kit (Qiagen, Hilden, Germany) and OligodT primers (Promega, Mannheim, Germany), starting from equal amount of RNA for each sample. Left ventricular tissues were processed using QIAschredder (Qiagen, Hilden, Germany). SYBR Green (Qiagen, Hilden, Germany) qRT-PCRwas performed using Qiagen Rotor-Gene machine. The sequences of primers used in this study and corresponding annealing temperatures are presented in Table1. RPL32 was used as a housekeeping gene for normalization of gene expression.

**Table 1:** List of primers used in the study

Gene	Sequence	Annealing temperature (°C)
ANP	Qiagen (QT00250922)	55
BNP	Qiagen (QT00107541)	55
Col.3	Forward: 5′-TGGTCCTCAGGGTGTAAAGG-3′ Reverse: 5′-GTCCAGCATCACCTTTTGGT-3′	
EGFP	Forward: 5′-AGTCCGCCCTGAGCAAAGA-3′ Reverse: 5′-TCCAGCAGGACCATGTGATC-3′	60
HSP70	Forward: 5′-CCCGGTGTGGTCTAGAAAACA-3′ Reverse: 5′-CCATGAAGAAGACTTTAAATAACCTTGAC-3′	57
RCAN1	Qiagen (QT01053430)	
RPL32	Forward: 5′-GGGAGCAACAAGAAAACCAA-3′ Reverse: 5′-ATTGTGGACCAGGAACTTGC-3′	55
SMIT	Qiagen (QT00341355)	55
TGF-β	Qiagen (QT00145250)	55

### Histological analyses

Hearts were fixed in 4% *p*-formaldehyde (PFA) overnight at 4 °C and embedded in paraffin prior to histological processing. For visualization of collagen fibers, sections were subjected to Masson’s Trichrome Staining (Sigma Aldrich, Munich, Germany). All sections were stained at the same time to avoid variation in staining intensity. Images were taken in random areas of the left ventricle using a brightfield microscope (Leica DM500, Leica Microsystems, Mannheim, Germany). Percentage blue area out of total section dimension was measured by ImageJ (1.51p, National Institute of Health, US) and further used for analysis.

### Cell size measurement

In vitro NRVCM area measurement was performed according to established procedures. Immunofluorescent staining of α-actinin (Sigma-Aldrich, Munich, Germany) was performed in cell preparations according to standard protocols and images were taken with 10 × magnification (BZ-9000 Keyence, Itasca, USA). Cardiomyocytes were identified to be positive for the stained marker, while fibroblasts, occasionally present in these primary cell cultures, were negative. A total of 200 cells were analyzed in each treatment group. Cell size was measured using BZ-II Analyzer (version 2.1, HybridCellCount module).

To determine cardiomyocyte cross section in the myocardium, frozen sections were subjected to cell membrane staining with fluorescently labeled WGA (Thermo Fischer Scientific, Bremen, Germany). Images were taken using a confocal microscope (Zeiss LSM 800, Oberkochen, Germany) and relative cell area was analyzed by ImageJ.

### Transverse aortic constriction

Animal experiments were carried out under the guidelines from Directive 2010/63/EU of the European Parliament on the protection of animals used for scientific purposes with approval of the regional authorities in Karlsruhe (G180/12) and Kiel (V312-7224.121-4). Animals were kept under standard conditions (Interfaculty Biomedical Facility, Heidelberg, Germany and the animal facility of the University Medical Center Schleswig–Holstein, Kiel) with 12-h light, 12-h night cycle; water and food was offered ad libitum. Transverse aortic constriction (TAC) was performed in 10 weeks old C57BL/6 N mice as previously described [26], with a 27-gage needle for stenosis induction. Successful ligation was confirmed by measuring the right carotid/left carotid flow velocity ratio. Echocardiography was performed at baseline, 2, 4, and 6 weeks (prophylaxis study) or at 2 and 6 weeks (therapy study) post TAC using a VisualSonics Vevo 2100 imaging system and the 40 Hz MS-550D micro scan transducer. The measurements and data analyses were performed by an experimenter blinded to the treatment. Long axis and M-mode short axis cine loops were recorded. Left ventricular ejection fraction (EF) and left ventricular posterior wall diameter at diastole (LVPW,d) were determined using VisualSonics software. Animals were sacrificed 6 weeks after surgery. Heart weight/tibia length ratio was measured as a marker of cardiac hypertrophy, as well as lung weight/tibia length ratio for monitoring heart failure induced lung edema. Body weights were recorded daily.

### NFAT activity assay

NFAT activity was analyzed using a TransAM® NFATc1 kit (Active Motif, Carlsbad, USA) according to the instructions provided by the manufacturer. A total of 15 μg nuclear extract was added to each well.

### Immunocytochemistry

Immunocytochemistry was used to detect NFATc1 translocation under pro-hypertrophic conditions. In brief, NRVCMs were fixed with 5% PFA for 5 min and incubated for 1 h in a buffer containing 3.5% BSA and 0.1% Triton X-100 to block non-specific binding of antibodies. Afterwards, cells were treated with primary antibody (Santa Cruz, Heidelberg, Germany) overnight at 4 °C in a humidified atmosphere. The corresponding fluorescent dye-labeled secondary antibody (Thermo Fischer Scientific, Darmstadt, Germany) was added after a series of washing steps and incubated for 1 h at ambient temperature. Images were quantified using ImageJ.

### Western blot analysis

Western blot analysis was conducted according to standard protocols. In brief, 20 μg protein was separated by SDS-PAGE and next transferred onto nitrocellulose membranes. Specific antibodies against tubulin (Sigma-Aldrich, T5168, dilution 1:10 000 in milk) and histone H3 (Cell Signaling, 4499, dilution 1:5000 in milk) were incubated overnight at 4 °C. Next, membranes were treated with horseradish peroxidase-coupled secondary antibodies (Dianova, dilution 1:10 000 in milk) for 1 h at ambient temperature. Detection was performed using ECL Detecton reagent (Thermo Fischer Scentific).

### Enzyme-linked immunosorbent assay

Enzyme-linked Immunosorbent Assay (ELISA) was performed according to the manufacturer´s instruction (Sigma-Aldrich, Munich, Germany), starting from 50 μL of plasma. Samples were diluted 1:1 with assay diluent prior to analysis.

### Statistical data analysis

Statistical data evaluation and generation of graphs were made using GraphPad Prism 7 software (San Diego, California, USA). Differences between 3 or more groups were assessed using One-way ANOVA, followed by a Tukey’s multiple comparisons test for particular pairs of groups. Mann–Whitney *U* test was used to compare two groups. All data were normalized to the respective control group. A *p* value < 0.05 was considered significant. Data are presented as means ± SD of individual experiments.

## Results

### Hairpin NFAT decoy oligodeoxynucleotides decrease ET-1-induced cardiomyocyte hypertrophy

**Fig. 1:**
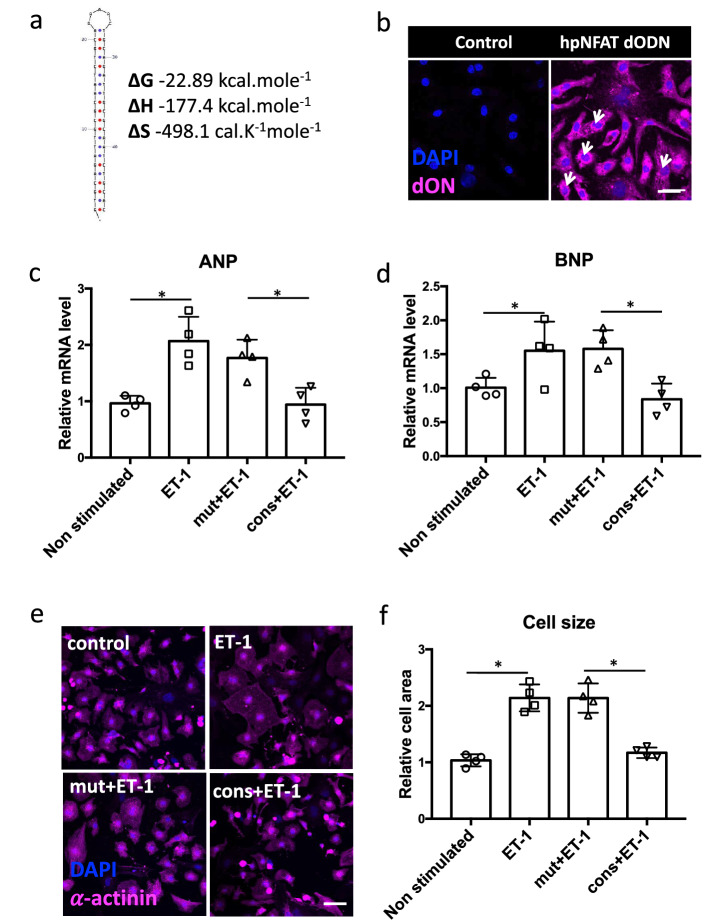
Hairpin NFAT dODNs exert anti-hypertrophic effect in NRVCMs. **a** Sequence of the designed NFAT dODN and physical properties, showing enhanced stability as a hairpin form. **b** Representative confocal microscope images showing the uptake of fluorescently labeled dODNs (magenta) by NRVCMs. Nuclei were stained with DAPI. Arrows point towards nuclear localization of dODNs. Scale bar represents 25 μm. **c**, **d** Statistical quantification of fetal gene products ANP and BNP on mRNA level. RPL32 was used as a housekeeping gene and values were normalized to non-stimulated cardiomyocytes as control. **e** Illustrative images of α-actinin detection by immunocytochemistry (magenta) of NRVCMs of the depicted treatment groups and **f** Statistical quantification of relative cardiomyocyte size as a measure of pro-hypertrophic response. Scale bar represents 25 μm. (*n* = 4, *p < 0.05, 20 images analyzed/group)

First, we designed a highly specific DNA-based dODN to neutralize NFAT activity, demonstrating remarkable stability in a hairpin loop conformation (Fig. 1a). This hairpin (hp) dODN is intended to neutralize all four calcineurin-dependent NFATs, i.e. NFATc1-c4 but not NFAT5, which is activated independently of calcineurin. Next, we confirmed that hairpin dODNs are taken up by neonatal rat ventricular cardiomyocytes (NRVCM) in vitro, and also translocate to the nucleus to some extent (Fig. 1b). To elucidate the effects of NFAT inhibition by this dODN on cardiomyocyte hypertrophy in vitro, NRVCMs were stimulated with ET-1 subsequent to pre-incubation with the consensus or mutant control dODNs. Real-time qPCR analysis indicated that NFAT neutralization with the consensus but not the mutant control dODN significantly decreased expression of the fetal genes ANP and BNP (Fig. 1c, d). Exposure to ET-1 also resulted in a marked increase in NRVCM size, which was virtually abolished by prior incubation with the consensus but not the mutant control dODN (Fig. 1e, f).

### AAV6-mediated hpNFAT RNA decoy oligonucleotide expression blocks ET-1-induced cardiomyocyte hypertrophy

**Fig. 2:**
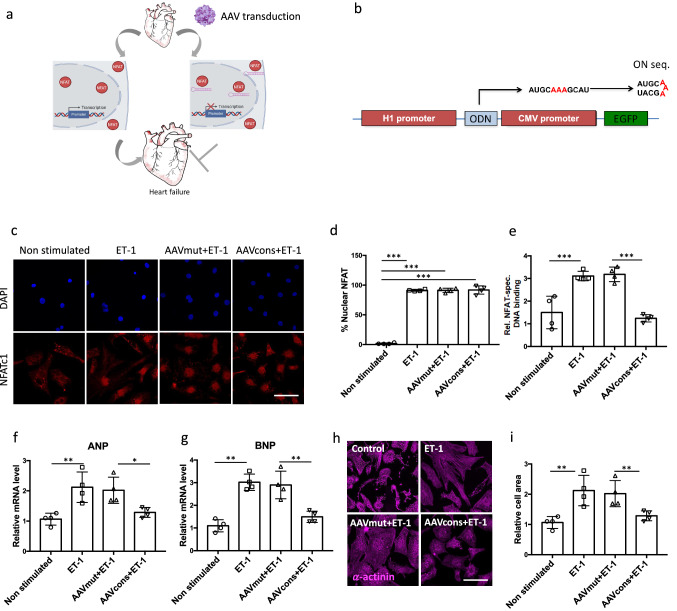
AAV6-mediated hpNFAT dONs delivery in NRVCMs reduces the pro-hypertrophic response to ET-1. **a** Graphical representation showing the project concept. Binding of members of the NFAT transcription factor family to specific promoter regions play a central role in the hypertrophic gene expression program (left). AAV-mediated cardiac expression of RNA-based dONs aims to neutralize NFATc1-4 and prevent its interaction with NFAT binding sites in the genome (right). **b** Schematic representation of dON generation following AAV transduction. Decoy ONs are highly stable as hairpin structure. ON seq: sequence of the designed NFAT dON. **c** Representative images showing NFATc1 (red) expression in NRVCMs in depicted treatment groups. DAPI served as a nuclear marker. Scale bar represents 25 μm. **d** Statistical quantification of nuclear NFAT abundance following ET-1 stimulation. **e** Determination of NFAT-specific DNA-binding capacity in nuclear extracts of NRVCMs. **f**, **g** Quantification of ANP and BNP expression levels following AAV6 transduction as markers of pro-hypertrophic response. **h** Representative immunocytochemistry images showing α-actinin staining (magenta). **I** Statistical quantification of cardiomyocyte cross-sectional area in frozen sections stained with WGA (*n* = 4, *p < 0.05, **p < 0.01, **p < 0.01 20 images analyzed/group)

Having demonstrated that neutralizing NFAT using hpNFAT dODN interferes with cardiomyocyte hypertrophy, we next aimed at developing this approach further into a continuous delivery system for the nucleic acid-based drug (Fig. 2a). For this purpose, we cloned the sequences of the designed consensus and mutant control hpNFAT dODNs downstream of the H1 promoter into an AAV plasmid backbone (AAVcons and AAVmut, *cf.* Figure 2b) and generated AAV serotype six vectors for in vitro studies. Next, we investigated nuclear translocation and binding of NFATc1, representative for NFATc1-4 as these NFAT family members share a conserved DNA-binding domain that specifies binding to the DNA core sequence (A/T)GGAAA [31]. While NFATc1 translocation to the nucleus of NRVCMs in response to ET-1 was unaffected (Fig. 2c, d), binding to its cognate *cis*-regulatory elements in the promoters of its target genes, as determined by an ELISA-based DNA-binding assay, was virtually abolished following AAV6cons but not AAV6mut transduction of the NRVCMs (Fig. 2e). Moreover, in AAV6cons but not AAV6mut-treated NRVCMs, ET-1-stimulated expression of the fetal genes ANP and BNP was nearly reduced to baseline (Fig. 2f, g) as was the ET-1-mediated increase in cell size (Fig. 1h, i), a parameter for the induction of hypertrophy in vitro. Collectively, these data verify that the RNA-based hpNFAT consensus dON is expressed by the cultured NRVCMs at a concentration that effectively neutralizes NFAT activity stimulated by a prototypic pro-hypertrophic mediator.

Specificity of the dON was further analyzed in vitro. NFAT5 plays a vital role in cardiomyocyte physiology by regulating the response to osmotic stress [1]. We therefore addressed the question whether the expressed dON neutralizes NFAT5 in cardiomyocytes and thus interferes with the expression of prototypic NFAT5 target genes. Accordingly, cardiomyocytes were exposed to 100 mmol/L LiCl for 24 h to upregulate NFAT5-dependent gene expression. Immunofluorescence analysis (Suppl. Figure 1a) revealed that neither the DNA-based hpNFAT consensus dODN nor AAV6-mediated expression of the RNA-based hpNFAT consensus dON influences NFAT5 translocation to the nucleus (Suppl. Figure 1b). Likewise, osmotic stress-induced expression of SMITS and HSP70, products of the prototypic NFAT5 target genes *SLC5A3* and *HSPA*, was not affected by the two consensus oligonucleotides (Suppl. Figure 1c, d).

### AAV9-mediated cardiomyocyte-specific hpNFAT RNA decoy oligonucleotide expression prevents pressure overload-induced cardiac hypertrophy and heart failure

**Fig. 3:**
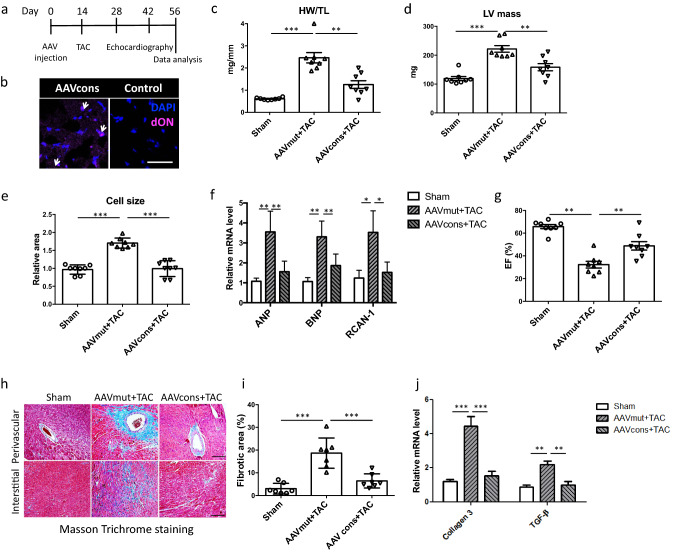
AAV9-mediated generation of hpNFAT dONs prevent TAC-induced myocardial hypertrophy and dysfunction in mice. **a** Graphical representation of experimental timeline in the preventive approach. **b** Illustrative confocal images proving efficient hpNFAT dON production in cardiomyocytes after tail vein injection of AAV9. Scale bar represents 25 μm. **c** Statistical quantification of heart weight/tibia length (HW/TL) of mice subjected to the depicted treatments. **d** Measurement of left ventricular (LV) mass by echocardiography following TAC. **e** Statistical quantification of relative cardiomyocyte cross-sectional area, measured after WGA staining on cardiac frozen sections. Values were normalized to the sham group. **f** Gene expression analysis of hypertrophy markers ANP, BNP and RCAN-1 on the mRNA level by qPCR. RPL32 served as a housekeeping gene. **g** Myocardial function measured by ejection fraction 6 weeks after induction of cardiac hypertrophy. **h** Illustrative images showing Masson Trichrome staining of cardiac sections of the groups as indicated, and **i** statistical quantification of the percentage of blue area, marking pathological extracellular matrix deposition (20 images analyzed/group, scale bar: 20 μm). **j** Relative expression levels of collagen 3 and TGF-β as a further confirmation of fibrosis development after TAC (*n* = 8, *p < 0.05, **p < 0.01, ***p < 0.001)

To validate the above results in vivo, AAV9 vectors expressing hpNFAT consensus or mutant control ON were intravenously injected in mice two weeks prior to induction of cardiac hypertrophy through TAC (Fig. 3a). This allowed expression of the RNA-based dON before induction of cardiac hypertrophy (preventive treatment approach). Successful generation of the dON was verified in an independent experiment by fluorescence in situ hybridization of cardiac sections. 2 weeks after transduction (Fig. 3b). While mice injected with AAV9 expressing the mutant control ON presented with significantly elevated cardiac hypertrophy 6 weeks post TAC as compared to sham-treated animals, this was markedly reduced in mice pretreated with AAV9 expressing the consensus dON (Fig. 3c–e, Suppl. Figure 2a,b). Moreover, pressure overload-induced upregulation of the cardiac pro-hypertrophic gene program in these animals was nearly abolished (Fig. 3f, Suppl. Figure 2c–e), and echocardiography revealed a significant improvement in cardiac function as well as a significant reduction in cardiac hypertrophy (Fig. 3g, Suppl. Figure 2f, g).

Fibrosis is a hallmark of heart failure and directly contributes to impairment of cardiac function by decreasing contractility and oxygenation [43]. AAV9-mediated expression of the mutant control ON prior to TAC resulted in a prominent increase both in perivascular and interstitial fibrosis as well as an increased expression of collagen 3 and transforming growth factor β (TGF-β), which are prototypic for pressure overload-induced replacement fibrosis (Fig. 3h–j). All of these TAC-induced changes were essentially prevented following pre-treatment of the mice with the AAV9 expressing the consensus dON, further emphasizing its striking efficacy.

### AAV9-mediated cardiomyocyte-specific hpNFAT RNA decoy oligonucleotide expression abrogates TAC-induced cardiac hypertrophy and heart failure

**Fig. 4:**
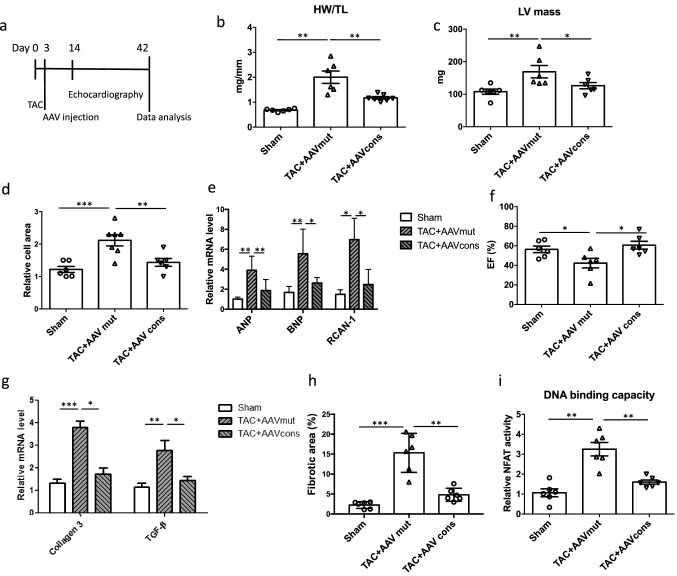
AAV9-mediated delivery of hpNFAT dONs rescues cardiac dysfunction due to pressure overload in mice. **a** Timeline showing the experimental design of our in vivo experiment. **b** Quantification of heart weight/tibia length (HW/TL) ratio as a parameter of cardiac hypertrophy in the respective treatment groups. **c** Echocardiography-based measurement of left ventricular mass 6 weeks after TAC. **d** Statistical quantification of relative cell area of cardiac sections stained with WGA in the mentioned treatment groups. Values were normalized to the sham group. **e** Gene expression analysis of hypertrophic markers ANP, BNP and RCAN-1 in the myocardium of treated mice. RPL32 was used as a housekeeping gene and values were normalized to sham-operated mice. **f** Analysis of myocardial function measured by ejection fraction 6 weeks after TAC. **g** Statistical quantification of extracellular matrix deposition by Masson Trichrome staining of heart Sects. (20 images analyzed/group, scale bar: 20 μm). **h** Relative expression levels of collagen 3 and TGF-β as markers of fibrosis. **i** Statistical quantification of NFAT activity following the specified treatments, 6 weeks after TAC (*n* = 6, *p < 0.05, **p < 0.01, ***p < 0.001)

The cardioprotective effects of AAV9-mediated expression of the hpNFAT consensus dON were further analyzed in a therapeutic experimental setting. For this purpose, mice were injected with AAV9 vectors harboring the consensus or mutant control ON 3 days after TAC, as depicted in Fig. 4a. As in the preventive experimental setting described above, TAC resulted in prominent cardiac hypertrophy, induction of the fetal gene program, systolic dysfunction, cardiac fibrosis and pro-fibrotic gene expression in the heart when the mice were treated with the mutant control ON expressing AAV9 3 days later (Fig. 4b–h, Suppl. Figure 3). In addition, there was a robust increase in nuclear NFAT activity in the cardiomyocytes 6 weeks after TAC (Fig. 4i, Suppl. Figure 4). Treatment with the consensus ON expressing AAV9 3 days post TAC essentially normalized all of the aforementioned parameters to the level in the sham-treated control animals, suggesting that AAV9-mediated expression of the hpNFAT dON is equally effective both in a preventive and therapeutic experimental setting.

Next, we aimed to investigate whether the described treatment affects NFAT target genes in non-target organs following AAV9 transduction. As expected for AAV9 vectors, we could detect EGFP expression primarily in the myocardium after systemic administration and to a lower extent also in skeletal muscle, liver, kidneys, and spleen (Suppl. Figure 5a). Interestingly, however, a decreased RCAN-1 mRNA level, indicative of the efficacy of the expressed dON, was only detected in cardiac muscle, as presented in Suppl. Figure 5b–d.

## Discussion

Our study provides a novel and effective therapeutic option for cardiac hypertrophy and heart failure, consisting of AAV-mediated delivery of a RNA-based dON neutralizing transcriptional activity of the NFATc1-c4 family of transcription factors. Following one-time systemic injection of the AAV9-based viral vector, the decoy oligonucleotide is specifically expressed by cardiomyocytes as a shRNA bearing the consensus binding site of NFATc1-c4, which potently down-regulates expression of NFAT-dependent pro-hypertrophic genes, hence preventing or inhibiting TAC-induced cardiac hypertrophy and subsequent heart failure. This novel therapeutic approach for delivery of dONs provides the opportunity to target specifically the myocardium, while avoiding non-specific adverse effects in other types of cells in the body.

Recent studies focusing on establishing the molecular mechanisms underlying cardiac hypertrophy have made it possible to advance new therapies and identify novel targets for gene therapy [6, 19]. Various transcription factors have emerged as critical players in the development/manifestation of this disease with various etiologies, making them attractive targets for gene therapy [5, 20, 35, 37, 44]. The NFAT family of transcription factors, except NFAT5, is an essential contributor to the progression of cardiac hypertrophy through governing changes in cardiomyocyte gene expression in response to a supra-physiological increase in wall stress (pressure overload) causing a maladaptive increase in cardiomyocyte size and rise in wall thickness ultimately leading to heart failure [36].

Several studies evidence the potential of DNA oligodeoxynucleotides as pharmacological tools to ameliorate symptoms of heart disease. Antisense ODNs directed against leukemia inhibitory factor (LIF) and cardiotrophin-1 were successful in downregulating angiotensin II-induced cardiac hypertrophy in vitro [40]. Moreover, a single systemic administration of antisense ODNs targeting phospholamban, a SERCA2a inhibitor, dramatically improved contractility in a pressure overload model of heart failure [34]. In addition, decreasing NF-κB target genes ameliorated cardiac remodeling associated with ischemia [23]. On the other hand, dODNs targeting transcriptional activity of GATA4 failed to ameliorate the endothelin-1 or phenylephrine-induced switch in cardiomyocyte phenotype in vitro, suggesting that disrupting the activity of this transcription factor is insufficient to observe a beneficial effect [38]. However, neutralization of GATA4 by administration of dODNs in vivo has not been validated. As the contribution of fibroblasts and vessels is increasingly acknowledged in cardiac hypertrophy, the role of distinct cardiac transcription factors, such as GATA4 or GATA6, needs to be considered also in non-cardiomyocytes [11]. Although NFAT plays a central role in cardiac hypertrophy, the approach described here may be tested on further transcription factor targets involved in induction of cardiac hypertrophy, such as the *MEF2*, *HAND*, *SFR* or *MITF* genes.

The pro-hypertrophic effects of NFAT family members c1 to c4 have already been well established in cardiomyocytes. NFAT activity has been demonstrated to be substantially increased in patients with dilated cardiomyopathy [10] and in various animal models of pathological hypertrophy [28, 45]. Furthermore, decreasing NFAT activity through administration of an inhibitory peptide (VIVIT) was shown to be an effective treatment for pressure overload-induced cardiac hypertrophy in a rat animal model preventing increased heart weight and serum concentrations of brain natriuretic peptide (BNP) and atrial natriuretic peptide (ANP) [24]. Although this approach may be therapeutically relevant, repeated injections of the peptide would be required in patients due to its relatively short half-life. Moreover, high doses of the peptide had to be administered to attain the observed therapeutic effect, which does not exclude the risk for possible off-target effects in other organs. Drug-based approaches of NFAT inhibition may not only result in potential extra-cardiac side effects, but might also be limited by distinct effects on NFAT subtypes. For example, the pharmacological NFATc3 inhibitor A-285222 protected from endothelial dysfunction in a murine diabetes model [13] but does not block NFATc2 playing a central role in pathological myocardial hypertrophy [2].

A particular requirement for any transcription factor neutralizing agent is that it should be specifically directed to the target transcription factor. The sequences of the dODN and dON used herein were designed according to the consensus DNA-binding site of NFATc1-c4 [39] to specifically neutralize only these NFAT family members but not NFAT5, which binds to distinct DNA motifs [25, 39]. As shown herein, expression of NFAT5 target genes upregulated through exposure to osmotic stress was not affected by the anti-NFAT1-4 dON, emphasizing the target specificity of the chosen nucleic acid-based drug candidate.

AAV-mediated expression of anti-NFAT1-4 dONs allowed a long-term cardiac effect of the therapeutic nucleic acid compound following single vector administration in our study similar to subcutaneous injection of the VIVIT peptide [2]. The NFAT-inhibiting peptide VIVIT was successfully expressed using an AAV-based approach in a mouse model of Alzheimer's disease [18]. However, no vector-mediated expression of VIVIT has been investigated in experimental cardiac hypertrophy so far.

Although AAV9 vectors mediated a predominant cardiac gene expression, some extra-cardiac transduction was also detectable. Nevertheless, we could not detect evidence of downregulation of NFAT target genes outside the heart. A potential reason could be that the expressed dONs may be solely active when nuclear translocation of NFAT is initiated as in cardiac hypertrophy and heart failure. Since TAC does not result in NFAT activation in non-cardiac tissue as observed in inflammatory liver diseases and fibrosis [3] or skeletal muscle growth and development [4], the hpNFAT dON overexpression may have no extra-cardiac side effects at least in our model.

In addition to reduced cardiac hypertrophy and improved cardiac function, we noted an almost complete prevention or inhibition of cardiac fibrosis as well as pro-fibrotic gene expression in TAC mice with AAV9-mediated expression of the hpNFAT dON. This result is in line with previous studies showing that NFAT inhibition through injection of FTY-720, a compound used for treatment of multiple sclerosis, reverses cardiac fibrosis in mice subjected to TAC. Moreover, TGF-β-induced collagen 1 and fibronectin production was shown to be NFAT-dependent [48]. Interestingly, fibronectin itself was proven to induce NFAT activity [21].

Although the data provided here clearly suggest a promising and innovative treatment option for cardiac hypertrophy transiting to heart failure, we acknowledge that there may be considerable differences between small animal models for heart disease and the situation in humans. For this reason, testing the feasibility of this approach in a non-rodent mammalian system is required. This would potentially constitute a step forward in bringing the suggested gene therapy closer to clinical trials and patients. Moreover, even though TAC is a well-characterized model for cardiac hypertrophy and heart failure, it is not without limitations and challenges with regard to translation into the human setting. This method causes a rapid induction of pressure overload, which might be different from gradual development of aortic stenosis in patients. In addition, mice subjected to TAC required rather early analysis to avoid loss due to increased mortality. Moreover, we have used young adult mice, which adapt faster to TAC than older animals [27]. In contrast, rat models of TAC present with a slower onset of left ventricular hypertrophy and disease progression [14]. Thus, as AAV vectors enable sustained cardiac gene expression, our approach should be studied regarding its long-term effects in additional models of cardiac disease involving abnormal activation of NFAT including pulmonary hypertension [7] or heart failure induced by myocardial infarction [45]. A further challenge for translation into the situation in patients is the requirement of choosing the right time point for vector application as repeated AAV application is limited by induction of neutralizing antibodies after the first injection.

In conclusion, our study is the first to show that a RNA-based dON targeting NFATc1-c4 can be generated in cardiomyocytes following AAV9 transduction, and can be employed both as a preventive as well as a therapeutic option for rescuing cardiac function in a pressure overload model in mice. Moreover, from a mechanistic point of view, our study undeniably unveils the importance of inhibiting cardiac hypertrophy to prevent transition into chronic heart failure.

## Supplementary Information

Below is the link to the electronic supplementary material.

Supplementary file1 (PDF 1335 KB)

## Data Availability

Any materials can be obtained from the authors upon request.
