# Supplementary material for: AAV-mediated expression of NFAT decoy oligonucleotides protects from cardiac hypertrophy and heart failure
Source: Basic Res Cardiol. 2021 Jun 4;116(1):38. doi: 10.1007/s00395-021-00880-w (PMC8178147; doi:10.1007/s00395-021-00880-w)
Supplement: Supplementary file 1 — Supplementary file1 (PDF 1335 KB) [file 395_2021_880_MOESM1_ESM.pdf]

Suppl. Fig. 1

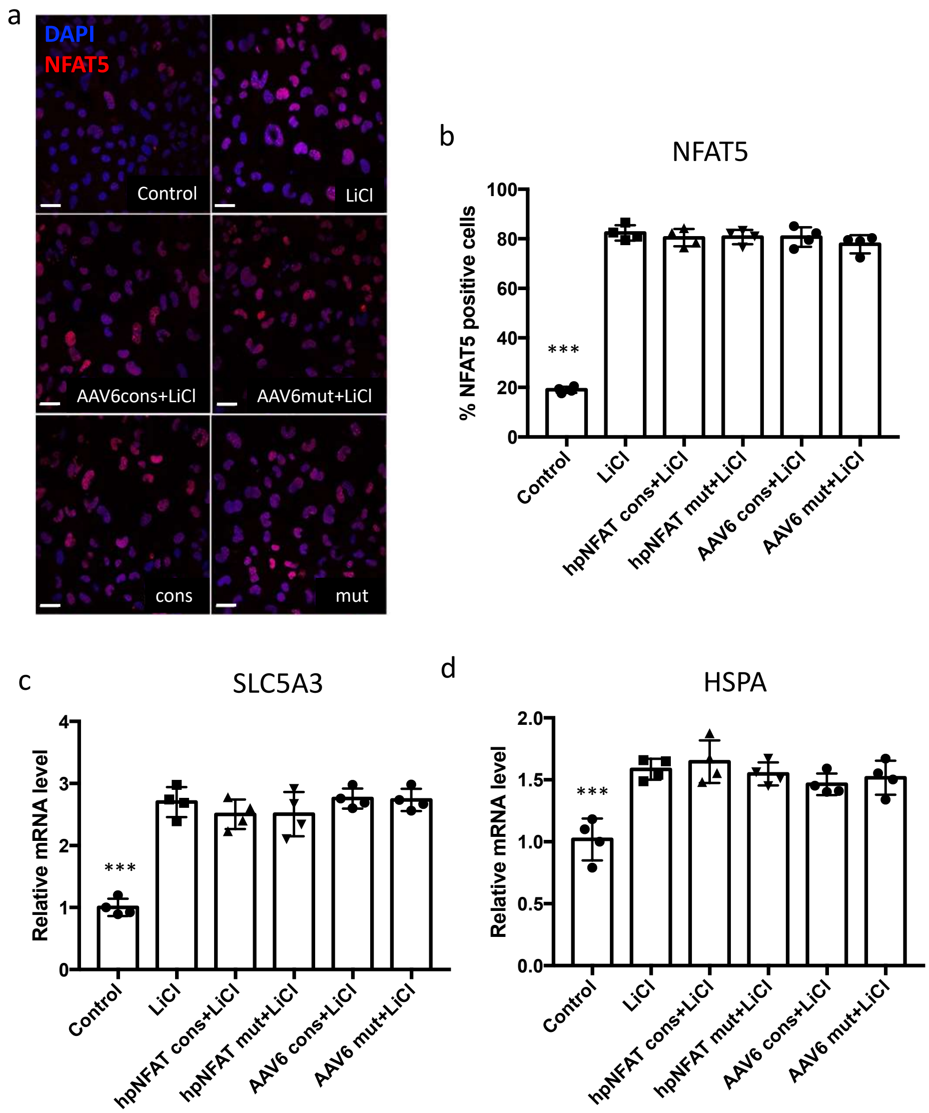

Suppl. Fig. 1. Hairpin NFAT dODNs do not affect NFAT5 protein level and activity in hyperosmotic stress conditions in HL-1 cells

(a) Illustrative images showing NFAT5 immunocytochemistry (red) in HL-1 cells treated with hpNFAT dODNs and exposed to LiCl. Nuclei were stained with DAPI (blue). Scale bar represents 25 µm. **(b)** Statistical quantification of NFAT5 nuclear translocation levels in the different treatment groups. **(c, d)** Gene expression analysis of NFAT5 target gene products SMIT and HSP70. Values were normalized to non-treated cells and RPL32 served as a housekeeping gene. Scale bar represents 25 µm (n=4, ***p<0.001, 20 images analyzed/group).

Supplementary Fig. 2

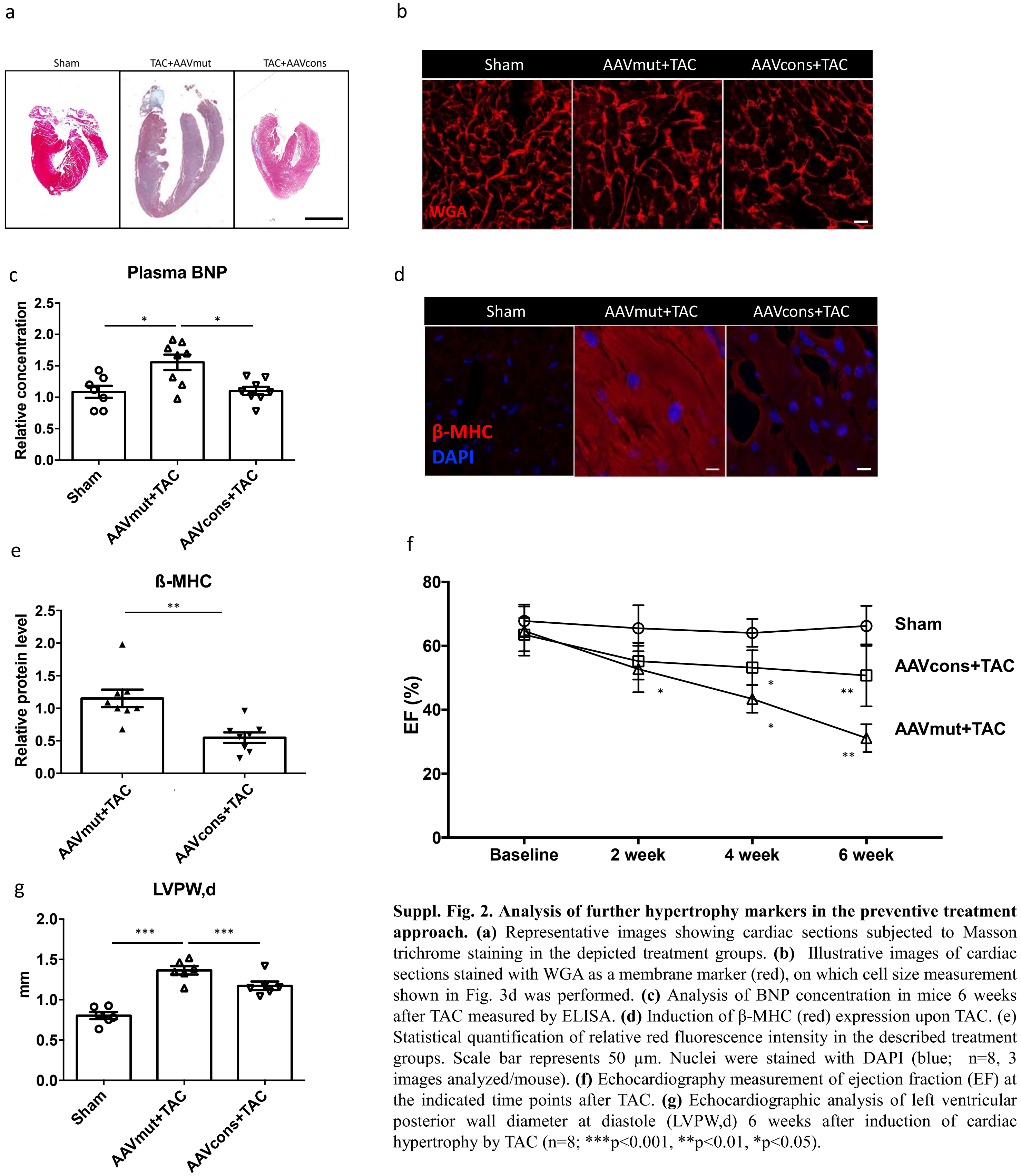

Suppl. Fig. 2. Analysis of further hypertrophy markers in the preventive treatment approach. (a) Representative images showing cardiac sections subjected to Masson trichrome staining in the depicted treatment groups. **(b)** Illustrative images of cardiac sections stained with WGA as a membrane marker (red), on which cell size measurement shown in Fig. 3d was performed. **(c)** Analysis of BNP concentration in mice 6 weeks after TAC measured by ELISA. **(d)** Induction of β-MHC (red) expression upon TAC. (e) Statistical quantification of relative red fluorescence intensity in the described treatment groups. Scale bar represents 50 µm. Nuclei were stained with DAPI (blue; n=8, 3 images analyzed/mouse). **(f)** Echocardiography measurement of ejection fraction (EF) at the indicated time points after TAC. **(g)** Echocardiographic analysis of left ventricular posterior wall diameter at diastole (LVPW,d) 6 weeks after induction of cardiac hypertrophy by TAC (n=8; ***p<0.001, **p<0.01, *p<0.05).

Supplementary Fig. 3

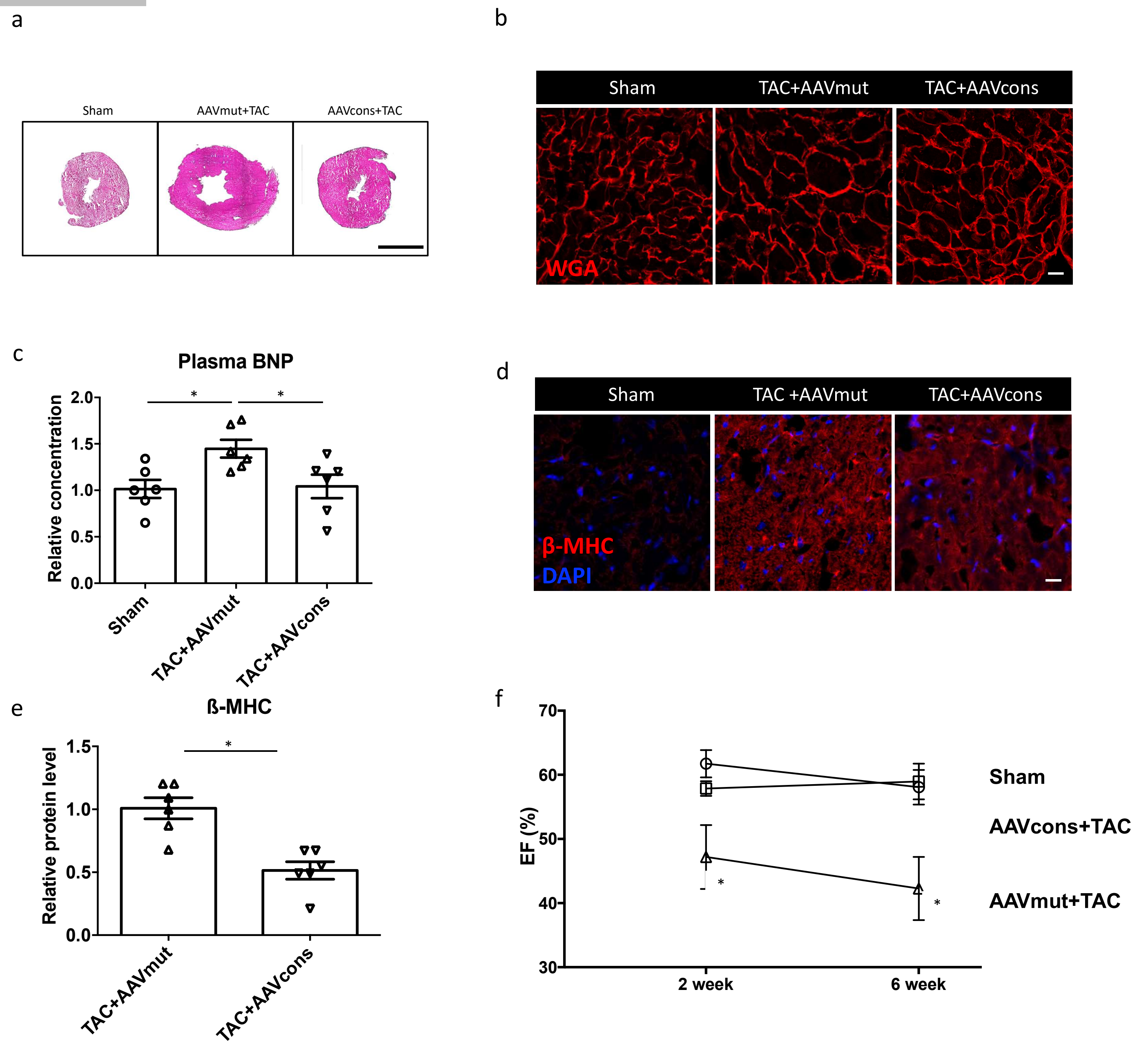

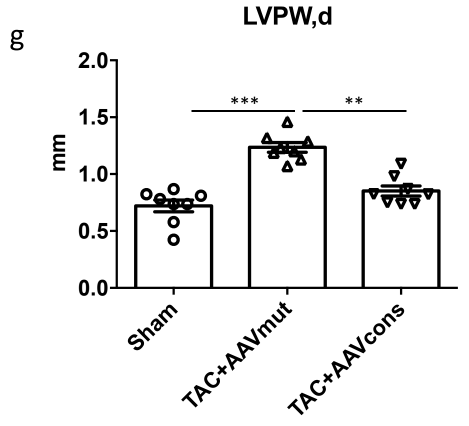

Suppl. Fig. 3. Analysis of additional hypertrophy markers in the therapeutic treatment approach. (a) Representative images showing Masson trichrome staining of cardiac tissue. **(b)** Illustrative images of cardiac sections stained with WGA as a membrane marker (red), on which cell size measurement shown in Fig. 4d was performed. **(c)** Analysis of BNP concentration in mice 6 weeks after TAC measured by ELISA. **(d)** β-MHC (red) expression detected by immunohistochemistry. **(e)** Quantification of relative red fluorescence intensity in myocardial tissue. Scale bar represents 50 µm. Nuclei were stained with DAPI (blue; n=6, 3 images analyzed/mouse). **(f)** Echocardiography measurement of ejection fraction (EF) at the indicated time points referring to the day of TAC in mice receiving gene therapy after TAC. **(g)** Echocardiographic analysis of left ventricular posterior wall diameter at diastole (LVPW,d) 6 weeks after induction of cardiac hypertrophy by TAC (n=6; ***p<0.001, **p<0.01, *p<0.05).

Supplementary Fig. 4

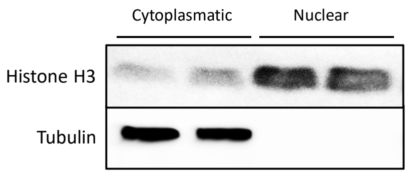

Suppl. Fig. 4. Subcellular fractionation of heart tissue on which NFAT binding capacity analysis was performed. Histone H3 served as a nuclear marker, while tubulin was used as an indicator of the cytoplasmic fraction.

Supplementary Fig. 5

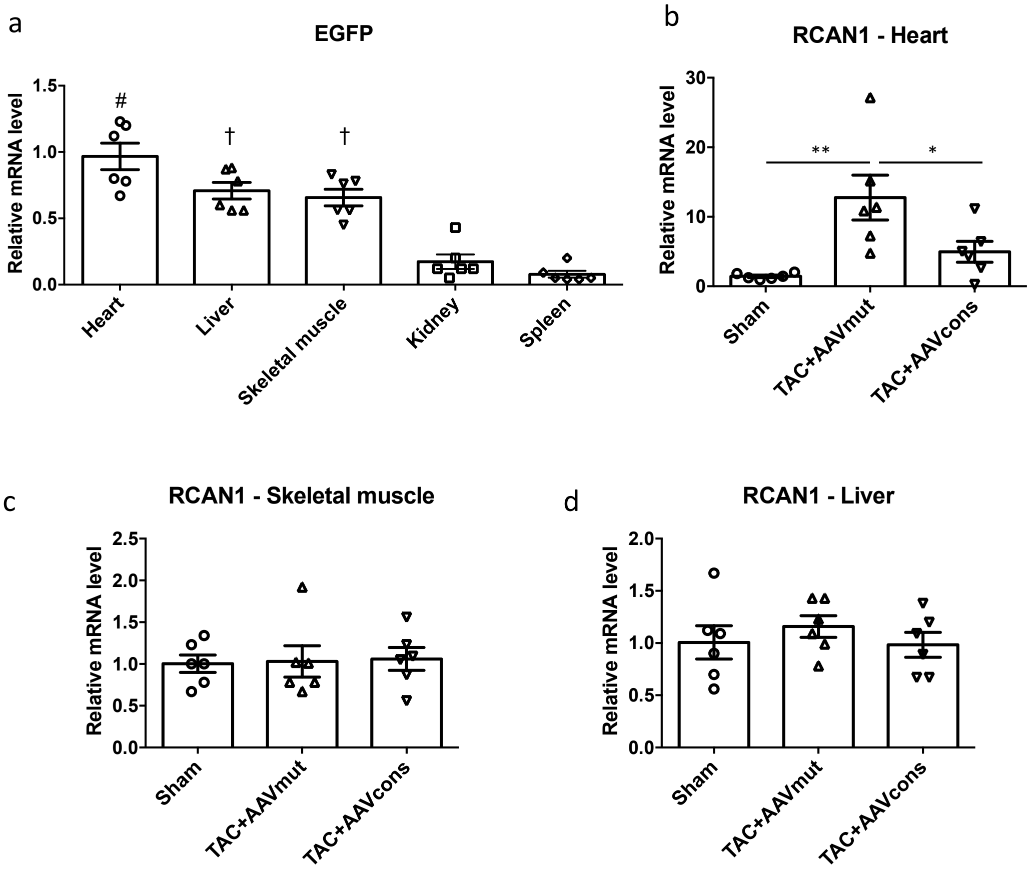

Suppl. Fig. 5. AAV9-mediated delivery of hpNFAT dONs does not affect RCAN-1 expression in non-target organs. (a) Statistical quantification of EGFP expression level in the depicted organs following systemic tail vein injection of AAV9 (# $p<0.05$ to liver and skeletal muscle, $p<0.01$ to kidney and spleen; † $p<0.01$ to kidney and spleen). Shown are RCAN-1 mRNA levels in **(b)** cardiac muscle, **(c)** skeletal muscle and **(d)** liver in mice subjected to TAC (* $p<0.05$, ** $p<0.01$).
